# The Effect of Aquatic Plant Abundance on Shell Crushing Resistance in a Freshwater Snail

**DOI:** 10.1371/journal.pone.0044374

**Published:** 2012-09-06

**Authors:** Johel Chaves-Campos, Lyndon M. Coghill, Francisco J. García de León, Steven G. Johnson

**Affiliations:** 1 Department of Biological Sciences, University of New Orleans, New Orleans, Louisiana, United States of America; 2 Laboratorio de Genética para la Conservación, Centro de Investigaciones Biológicas del Noroeste, La Paz, Baja California Sur, México; California State University Fullerton, United States of America

## Abstract

Most of the shell material in snails is composed of calcium carbonate but the organic shell matrix determines the properties of calcium carbonate crystals. It has been shown that the deposition of calcium carbonate is affected by the ingestion of organic compounds. We hypothesize that organic compounds not synthesized by the snails are important for shell strength and must be obtained from the diet. We tested this idea indirectly by evaluating whether the abundance of the organic matter that snails eat is related to the strength of their shells. We measured shell crushing resistance in the snail *Mexipyrgus churinceanus* and the abundance of the most common aquatic macrophyte, the water lily *Nymphaea ampla*, in ten bodies of water in the valley of Cuatro Ciénegas, Mexico. We used stable isotopes to test the assumption that these snails feed on water lily organic matter. We also measured other factors that can affect crushing resistance, such as the density of crushing predators, snail density, water pH, and the concentration of calcium and phosphorus in the water. The isotope analysis suggested that snails assimilate water lily organic matter that is metabolized by sediment bacteria. The variable that best explained the variation in crushing resistance found among sites was the local abundance of water lilies. We propose that the local amount of water lily organic matter provides organic compounds important in shell biomineralization, thus determining crushing resistance. Hence, we propose that a third trophic level could be important in the coevolution of snail defensive traits and predatory structures.

## Introduction

Anti-predator mechanisms are common in animals and occur in many forms. Animals can reduce the risk of predation through changes in behavior, life history, morphology, and chemistry [1]–[3]. While it is well established that diet plays a fundamental role in determining the effectiveness of chemical defense [4]–[6], the effect of diet on the effectiveness of morphological defenses has received less attention [3], [7]–[9]. In this study, we evaluated the relationship between the abundance of a primary food source, aquatic macrophytes, on shell crushing resistance in a freshwater snail that co-occurs with a specialized snail crushing fish.

Aquatic snails are excellent model systems to study anti-predator mechanism given their long history of interaction with shell-breaking predators such as crabs and fish [7], [9]–[11]. Under intense predation, snails can develop thicker shells, modify the structure of the shell material, and change the shape of the shell to reduce their probability of being predated [7], [12]–[15]. These processes are related to food intake in at least some snails [9], [16]. Because calcium carbonate is deposited at a constant rate, if snails reduce food intake (e.g. to avoid predators) they also grow more slowly and develop thicker shells, which can be more resistant to crushing [9], [17]. This metabolic process can be limited by calcium availability [18] but also by the availability of organic material [16]. Although most of the shell material in mollusks is composed of calcium carbonate, there is a small organic fraction which constitutes the skeletal organic shell matrix, which is more metabolically demanding to produce than the crystallization of calcium carbonate [19], and may therefore constrain the production of mechanically superior shells [16]. In agreement with all the findings presented above, recent experimental evidence suggests that the thickening of shells induced by reduced growth in situations of high predation risk is caused by the rapid deposition of mechanically weak and energetically inexpensive material with low organic content [9].

It remains to be demonstrated whether snails only develop stronger shells through the rapid production of shell material low in organic content. The conclusion that snails develop thicker shells more resistant to predation by reducing food intake has been reached in captivity, after subjecting snails to either starvation or low-food treatments [9]. In the field, snails may produce stronger shells by feeding on food sources that allow them to increase the organic content in their shells. The small organic fraction found in shells controls the formation of calcium carbonate crystals and play key roles in determining their properties [20]–[23]. Furthermore, the deposition of calcium carbonate in the shell is affected by the ingestion of organic compounds [24]. Freshwater snails feed on organic matter derived from aquatic vegetation and/or on microbes that feed on that organic matter [25]–[27], which suggests that shell strength may be affected by the abundance of organic matter in the environment. This hypothesis generates several predictions that to our knowledge have not been addressed in the context of resistance to predation. The simplest one is that under similar levels of predation and similar feeding and growth rates, snails living in environments richer in organic compounds important in shell biomineralization would develop stronger shells than snails living in environments limited in such organic matter, because the former would be able to add more organic material to their shells, improving their mechanical quality [16]. The availability of key organic compounds, as a food source, may also affect growth rates and therefore shell thickness. Predation intensity should also mediate the interaction between organic matter availability and growth rate (by inducing slower growth rates), making it difficult to predict patterns of shell strength in natural environments. However, if the abundance of organic matter rich in key organic compounds for shell biomineralization is a major factor determining shell strength, it is possible to predict a direct relationship between these two variables. Here, we investigate for the first time to our knowledge the potential interaction between predation intensity, organic matter abundance, and snail shell strength in the field.

Our study species is the small (<8 mm long) Mexican banded spring snail, *Mexipyrgus churinceanus* (Hydrobiidae) [28]. This freshwater snail and its specialized fish predator *Herichthys minckleyi* are endemic to the isolated Cuatro Ciénegas valley in northeastern Mexico and co-occur in the bodies of water that exist in the valley [29], [30]. The fish *Herichthys minckleyi* shows two pharyngeal jaw morphologies: small muscles and pointed teeth in “papilliforms” and robust muscles and enlarged flattened teeth in “molariforms” [31]. Papilliforms are incapable of crushing snails, while molariforms frequently crush and digest *M. churinceanus*. Crushing resistance very likely influences fitness in this snail. Only the snails with stronger shells survive predation once captured by a fish; the snails become virtually invulnerable to crushing predation when they reach their upper shell length range at about 7 mm [31], [32].

Shell crushing resistance in *M. churinceanus* varies geographically, and it is not explained by geographic or genetic distance among populations; it is correlated with the relative frequency of molariform *H. minckleyi* [29], [30]. The correlation between molariform frequency and snail crushing resistance in Cuatro Ciénegas is negative, which has been interpreted as a consequence of the relative disadvantage of molariforms in areas where snail crushing resistance exceeds the crushing capabilities of the fish [30]. Other biotic factors may contribute to explain the variation in crushing resistance among populations [30]. For instance, the local abundance of aquatic macrophytes may influence crushing resistance in Cuatro Ciénegas according to a previous study [29]. The authors of that study noted that the abundance of the most common aquatic macrophyte in the area, the native water lily *Nymphaea ampla* [33], varied greatly among bodies of water. Furthermore, water lilies were abundant in areas where crushing resistance was high and rare in areas where it was low [29]. Based on this observation, we hypothesized that organic compounds that are essential to shell strength and are not synthesized by the snails must be obtained in the diet, specifically from detritus containing water lily organic matter. This could explain why variation in shell strength is influenced by the abundance of organic matter derived from aquatic macrophytes [30]. In the current study, we test this pattern by evaluating whether local variation in crushing resistance in *M. churinceanus* is related to the local abundance of *N. ampla* (hereafter the water lily). Although crushing resistance might be related to the abundance of other, less common aquatic macrophytes, we expect it to respond more strongly to the abundance of the water lily because it is the most common species in the area [33].

Hydrobiid snails are believed to feed primarily on detritus and bacteria [34], [35]. We conducted an analysis of stable isotopes to test the assumption that *M. churinceanus* feeds directly on water lily detritus and/or on the substrate bacteria that feed on water lily detritus. We assumed that this detritus contains organic compounds essential for shell strength. The isotopic ratio values for carbon (C) and nitrogen (N) are commonly used to reconstruct the diet of animals, including snails [26], [36]. This type of analysis is based on two key findings. First, different food sources/taxa usually have different isotopic signatures [37], [38]. Second, it is possible to estimate the trophic position of species within food webs using their isotopic signature [38]. The ratio of C^13^ to C^12^ (i.e. δ^13^C) in a consumer species is similar to or slightly higher than the δ^13^C in the food the species eats (difference: −1 to 1‰), while the ratio of N^15^ to N^14^ (δ^15^N) is 2 to 5‰ higher in consumers in relation to their food, i.e. a single trophic step [39], [40]. Hence, if *M. churinceanus* eat water lily detritus directly, then the δ^13^C values in *M. churinceanus* tissue should match the δ^13^C values of live lily tissue (±1‰), and the δ^15^N in *M. churinceanus* should be 2–5‰ higher compared to live lily tissue (i.e. one trophic step). However, the isotopic signature can be very different if they only or also eat the bacteria that grow on water lily detritus.

Several studies on freshwater streams and lakes have reported that invertebrates that feed on detritus do not match the δ^13^C of that detritus; their δ^ 13^C signature is significantly reduced compared to the detritus [26], [39]. McGoldrick et al. (2008) demonstrated experimentally that the assimilation of ^13^C depleted bacteria that have grown on detritus is the simplest explanation for these reports. The rationale behind this conclusion is that both chemoautotrophic and heterotrophic bacteria found on bottom sediments can discriminate strongly against ^13^C, which results in a decrease of 3–5‰ in their δ^13^C signature compared to that of the substrate where they feed; this signature is then matched by the invertebrates that consume the bacteria [26], [39]. Similar reductions in δ^15^N between substrate and invertebrate consumers may also occur in some species [39], [41] but not others [26]. In the latter case, the typical 2–5‰ increase in δ^15^N between aquatic invertebrates and the substrate where they feed is expected even if they consume ^13^C depleted bacteria [26].

*Mexipyrgus churinceanus* is generally found only in soft sediment [28], so we measured δ^13^C and δ^15^N in the sediment to assess the possibility that snails consume C and N derived from water lilies that have been metabolized by sediment bacteria. Although bulk sediment likely includes a portion of organic matter that has not been metabolized, it should be possible to detect a net reduction in δ^13^C in the sediment compared to water lily tissue if bacteria are abundant and discriminate against ^13^C. If sediment bacteria feed on water lily tissue in the sediment, the δ^13^C in the sediment should be 3–5‰ lower than the δ^13^C in live water lily, although the decrease may be smaller or nonexistent depending on the amount of unmetabolized organic matter present in the sediment (i.e. it would be closer to the signature of water lily organic matter or any other organic matter present in the sediment). If *M. churinceanus* feeds on sediment bacteria then we predicted that the δ^13^C signature in *M. churinceanus* tissue should be close to the δ^13^C signature in the sediment. In the case of δ^15^ N, it is possible to predict either a decrease or an increase between water lily tissue and the sediment. A 3–5‰ decrease can be expected if bacteria discriminate against ^15^N; the decrease can be smaller or nonexistent depending on the amount of unmetabolized organic matter in the sediment. A 2–5‰ increase can be predicted if bacteria do not discriminate (i.e. one typical trophic step). In either case, an increase in 2–5‰ δ^15^N is predicted between the sediment and snail tissue if snails eat the bacteria present in the sediment (another trophic step).

After confirming that snails consume water lily organic matter metabolized by sediment bacteria, we tested whether the local abundance of water lilies is related to local levels of shell crushing resistance. We predicted a positive relationship between population crushing resistance and population water lily abundance. We also predicted that this relationship would be stronger than any other relationship between crushing resistance and any potential confounding factor. Altogether, these predicted results would indirectly suggest that the abundance of water lily organic matter, which presumably include organic compounds important for shell strength, is a main factor determining shell strength.

As mentioned, we took into account the potential effect of other biotic and abiotic variables when we tested for potential relationships between water lily abundance and crushing resistance. The concentration of dissolved calcium (Ca) in water has been shown to affect crushing resistance in snails [18], so we included this variable in our analyses. The concentration of Ca in Cuatro Ciénegas is very high [42], [43] and therefore it should not limit shell crushing resistance in this area. For this reason, we predicted no relationship between crushing resistance and Ca concentration. We also measured the concentration of phosphorus (P) in the water because Cuatro Ciénegas is extremely depleted in this element [44] and therefore snail growth may be limited by its availability [45]. Because growth rates affect shell thickness and therefore crushing resistance [9], the effect of P concentration on crushing resistance is not straightforward to predict. Moreover, the local abundance of water lilies may be affected by the concentration of P [46]. Hence, snails may grow at a slower rate in environments poor in P and organic matter, producing thicker but relatively less resistant shells (inexpensive shell material). On the other hand, snails may grow thinner but stronger shells in environments rich in both P and organic matter (expensive shell material). For this reason, we tested for a potential positive correlation between P concentration and crushing resistance, and between water lily abundance and P concentration.

An additional confounding factor is snail density. High water lily abundance could cause higher snail densities due to more food resources, and higher snail densities can decrease growth rates among individuals due to intraspecific competition. Hence, if lower growth rates result in thicker, stronger shells, then any observed pattern could be driven directly by snail density. To evaluate this possibility, we tested for potential correlations between water lily abundance and snail density, and between snail density and crushing resistance. Finally, we considered the potential effect of water pH on crushing resistance because this variable can be related to the concentration of Ca in the water and can affect the rate at which snail shells dissolve naturally, but should not affect calcification rates [47], [48].

## Methods

### Ethics Statement

All necessary permits were obtained for the described field studies. The Mexican government provided the research permits SGPA/DGVS/02680/10 and DGOPA.04701.160610.- 2783 to conduct this study in the Cuatro Ciénegas protected area.

### General Procedures

We sampled water lilies (abundance and tissue for isotope analysis), fish density, snails (density, crushing resistance and tissue for isotope analysis), water (to determine pH and the concentration of Ca and P), and the soft sediment where snails live (for isotope analysis) from ten localities (pools and rivers) nested within the three main natural drainages that exist in the valley of Cuatro Ciénegas (Figure S1, Table S1). All sampling was conducted in August 2010, except for water lily abundance, which was conducted in July 2009 and repeated in August 2010. Due to logistic limitations, the abundance of water lilies in the Pozas Azules site was only sampled in June 2009. The effects of hurricane Alex, which affected the study area in July 2010, prevented us from sampling additional sites.

Because we noticed changes in water lily abundance between years in some pools, we only used the data from 2010 to analyze the current relationship between crushing resistance and water lily abundance. Substrate bacteria show the isotopic signature of a single trophic step within hours after feeding on a specific substrate [39] while snails take a few months [36], [49]. Hence, we only analyzed the isotopes in samples collected in the same year (August 2010) to evaluate whether snails feed upon organic matter derived from water lilies.

### Water Lily, Sediment and Water Samples

We estimated the abundance of water lilies in quadrats (1×1 m each) located on transects systematically placed in the bodies of water where the snails were collected. In large bodies of water such as rivers, a transect was placed at the point where we first reached the site. The transect crossed the entire body of water, from shore to shore if the width of the body of water was shorter than 37 m, which was the maximum length of the transect. Four additional transects were set parallel to the first one, 30 m away from each other (2 on one side of the first one, 2 on the other side). In smaller bodies of water (i.e. pools), we set only 3 parallel transects evenly spaced from each other. One transect was set in the middle of the pool and the other two on both sides of the middle one. The transects usually crossed the pool from side to side in these cases. We counted the presence or absence of water lilies in each quadrat. We then calculated the proportion of quadrats with water lilies per transect.

In addition, in each site we collected three live water lily tissue samples for isotope analysis. The samples were collected on three different transects, from a random quadrat within each transect. We also took three sediment samples per site for isotope analysis and three water samples for Ca and P analysis, using the same method. The pH of each water sample was measured in the field with a portable pH meter (Oakton pHTestr 30). Due to logistical problems we could not take water samples from the Los Remojos site.

### Fish Density

In each site, we estimated the density of *H. minckleyi* fish in quadrats (9×9 m each) located randomly on either side of at least some of the transects described above. Four quadrats per site were used in small pools, while 8 quadrats per site were used in rivers and large pools. In each quadrat, two observers (the same throughout the study) located on opposite corners simultaneously counted the maximum number of fish detected at the same time during a period of five minutes. The two counts where averaged for each quadrat. After the counts were finished, we used gill nets to catch *H. minckleyi* fish with the purpose of estimating the proportion of molariforms and papilliforms in each population. The morphotype of each individual was determined in the field by recording the presence or absence of the conspicuous molariform teeth on the lower pharyngeal jaw using a portable otoscope [32]. We caught between 20 and 30 individuals in all sites, except for Juan Santos (n = 16) and Tío Cándido (n = 7). We then estimated the density of molariforms in each site by multiplying the proportion of molariforms in each site by the average density of individuals at that site. All densities were expressed as number of fish/m^2^. We did not measure the density of fish in the Pozas Azules pool because it was very small and only contained a few adult fish.

### Snail Samples

Individuals were collected randomly along the transects described above for each location. In total, thirty individuals were collected for each of the ten locations, except for Tío Cándido (n = 24), Escobedo (n = 25), and Río Mesquites (n = 31). Snails were collected in quadrats of known area, which allowed us to obtain density estimates for all sites. The snails were placed in water and transferred to a laboratory in Cuatro Ciénegas to measure shell resistance to crushing within a few hours. Crushing resistance scales with size in this species [29], [30], [32], so we measured the length of each shell to correct crushing resistance by shell length when populations were statistically compared. Shell length was measured to the nearest 0.1 mm from the apex to the bottom of the aperture from pictures taken with a microscope, using the program tpsDig 2.14 [50]. The snails were then crushed between two force plates of a Chatillon DFM50 force gauge with an automated Chatillon LTMCM-6 stand. The mobile force plate was set at 2.54 cm/min crushing speed. The force in Newtons needed to crush the snail at the time of shell failure was recorded for each snail. Tissue samples (mostly foot tissue) were kept for isotope analysis. It was necessary to combine the tissue samples from five snails to have enough material for isotope analysis, so we used 5 snails from three transects to obtain 3 replicates per site.

### Ca and P Analysis

Following standard procedures [51], water samples (100 ml) were filtered in the field immediately after collection using cellulose acetate membrane filters with a pore of 0.45 µm (Whatman, 47 mm in diameter), and preserved with nitric acid for posterior analyses of metals at the University of New Orleans. We determined the concentration of Ca and P in the samples using a Varian Vista-MPX CCD-Simultaneous Induced Coupled Plasma-Optical Emission Spectrometer. The machine was calibrated for each element through dilutions of commercial standards: 1000 ppm Calcium Reference Standard (Fisher Scientific) and 1000 pm Phosphorus AA Standard (Ricca Chemical Company). Each one of the three replicates per site was measured 3 times, and an average was calculated for each replicate. All samples were analyzed within a period of six months after collection.

### Isotope Analysis

All samples were dried in an oven at 60°C for 48 hours immediately after being collected. Samples were then sieved to remove shell fragments (in the case of sediment), and ground using mortar and pestle. Samples were commercially analyzed at the Colorado Plateau Stable Isotope Laboratory (Northern Arizona University). In this lab, the samples were fumigated with acid to remove carbonates, dried, ground again, weighed and encapsulated for analysis. At least 0.9 mg of each sample were analyzed for δ^13^C and δ^15^N in continuous-flow mode using a Thermo-Finnigan Deltaplus Advantage gas isotope-ratio mass spectrometer interfaced with a Costech Analytical ECS4010 elemental analyzer. The data were normalized using internationally-accepted isotope references standards: IAEA-CH6, CH7, N1, and N2. The δ^13^C and δ^15^N data were expressed relative to the international reference standard Vienna Pee Dee Belemnite for C, and air for N.

### Statistical Analyses

We used Analysis of Variance (ANOVA) to confirm inter population variation in water lily abundance, after confirming the assumptions of the test. In the case of shell crushing resistance, we used Analysis of Covariance (ANCOVA) to determine if this variable was significantly different among populations when adjusted by shell size. Although other methods are preferred over ANCOVA to compare morphological traits among populations [52], those methods were developed for multivariate measures of body size, which is not the case of this study. ANCOVA remains an acceptable method when there is only one response variable and one measure of body size as in this study, as long as there is homogeneity of slopes among populations [52], which we confirmed (Table S2). These and subsequent tests were conducted in R 2.13.0 [53] unless stated otherwise.

We conducted a trophic analysis with the isotope data to evaluate whether the snails were ingesting water lily organic matter. We first calculated one average δ^13^C value per site and one average δ^15^N value per site for each sample type. We then compared the average δ^13^C values of water lilies with those of the respective sediment samples across sites, and the latter with the respective values from snails. We used paired t-tests for all comparisons after confirming the assumptions of the test. The average difference between paired variables was calculated in the process. We did the same for δ^15^N.

Due to logistic limitations, our trophic analysis did not include other possible sources of organic matter that may have an isotopic signature roughly similar to that of water lilies, such as side vegetation or algae. In order to determine if water lily organic matter contributed to the isotopic signature of the sediment, we conducted a correlation analyses between the signature in water lily tissue and the signature in the sediment. This analysis gave us an idea of how much of the local variation in the isotopic signature in the sediment corresponds to variations in the isotopic signature of water lily tissue. We conducted the analysis for C and N separately. A significant correlation would indicate that at least a part of the signature in the sediment is the product of metabolization of water lily organic matter by sediment heterotrophic bacteria (as long as the difference in isotopic signature between water lily and sediment is consistent with the predicted values of a single trophic step).

We tested for a potential relationship between crushing resistance and water lily abundance, molariform density, P and Ca concentration, pH and snail density, and some combinations of the last variables by means of multiple linear regression and pairwise correlation analysis (after confirming the assumptions of normality and linearity). With a sample size of 9 or 10 sites, the power to detect a significant correlation or linear regression is very low unless the correlation coefficient is very strong (e.g. 0.9). For instance, the power to detect a correlation of 0.6 is close to 40% with α = 0.05, and the power to detect a correlation of 0.4 is close to 20%. We used two alternative approaches to circumvent this issue. First, we used randomization tests to determine the significance of pairwise correlation coefficients [54]. Second, we used the Akaike Information Criterion (AIC) to compare linear multivariate regression models that included several sets of the variables that may explain variation in crushing resistance [55], [56]. The AIC is used to compare models according to their fit to the data and complexity [55]. The model with the lowest AIC value is considered the best; however, models that have similar AIC values (≤6 units) to the best one should be regarded as the “confidence set” of models that are supported by the data, while models that have much higher AIC values (>7 units) than the best model have little or no support [56], [57]. Multiple regression models were inspected for multicollinearity (i.e. correlation among explanatory variables that can produce spurious interpretations regarding the contribution of each variable in the model [58]) by means of calculating the Variance Inflation Factor (VIF) of each term in the model; VIFs around 10 or higher imply serious problems of multicollinearity [59]. When multicollinearity was detected, pairwise correlation coefficients (Pearson) were calculated among the explanatory variables to identify what variables were correlated with each other. Once a pair of correlated variables was identified, partial correlations were used to determine which one of the two explanatory variables was more correlated with crushing resistance when the other explanatory variable was held constant [58]. The explanatory variable that was less correlated with crushing resistance was then removed from the model to eliminate that source of multicollinearity. We also used partial correlations to determine what variable in the “confident set” of models is more correlated with crushing resistance when the others are held constant.

The correlation and regression analyses were conducted using average values for each site. Size-adjusted crushing resistance values were obtained by fitting a linear least squares regression of shell length versus crushing resistance to generate “sized-adjusted” residuals. The residuals were then averaged by site. Using regression residuals is valid in this case because the scaling relationship between size and shell strength does not differ among locations [52], i.e. there is homogeneity of slopes among populations (Table S2). In the case of randomization tests, we randomized the dataset (independently for each variable) and calculated a correlation coefficient (Pearson) between the two randomized variables (i.e. a random coefficient). The procedure was repeated 100,000 times to generate a distribution of random coefficients. We then calculated the correlation coefficient between the two observed datasets (i.e. the observed coefficient) and determined the position of the observed coefficient within the distribution of random coefficients. The significance of the observed coefficient was calculated as the number of random coefficients that were equal or more extreme (i.e. larger or smaller according to the sign of the correlation coefficient) to the observed one, divided by the total number of random coefficients. The randomization tests were implemented in the program PopTools [60].

## Results

### Inter Population Variation in Crushing Resistance and Water Lily Abundance

Water lily abundance varied greatly among sites (F = 18.9, df = 8, 28, P<0.0001), ranging from sites with no water lilies to sites where >80% of the quadrats had the plant (Table S3). Crushing resistance scaled strongly with shell size (Figure S2) and varied greatly among sites when shell size was included as a covariate (F = 10.4, df = 9,278, P<0.0001, Table S2, Table S4).

### Trophic Analysis

**Figure 1:**
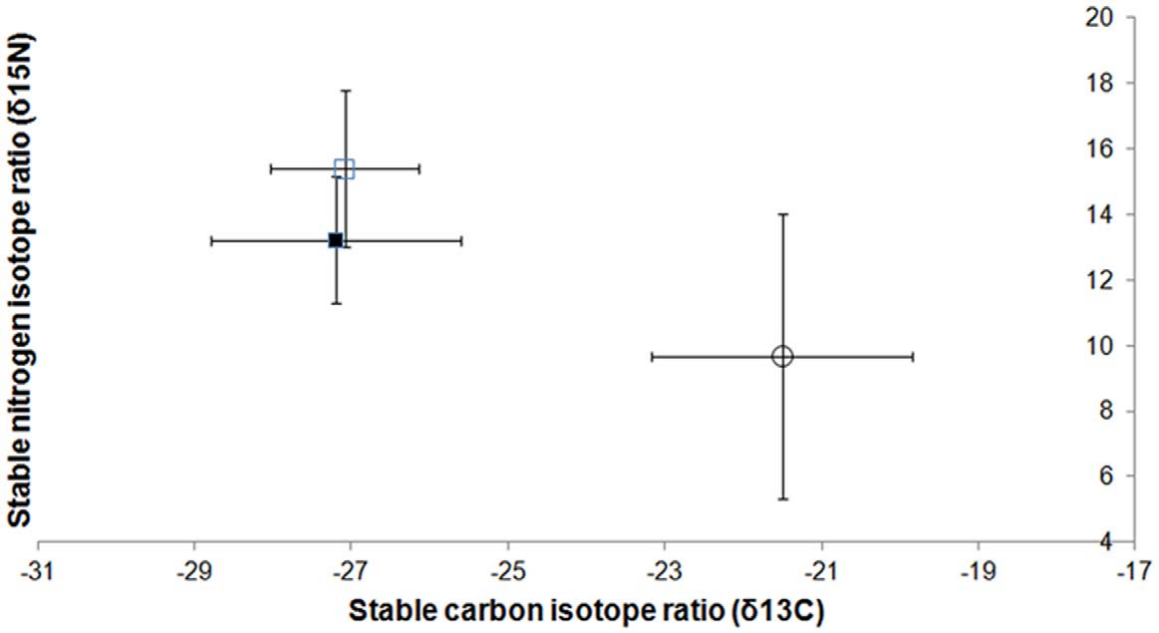
Stable carbon and nitrogen isotope ratios for samples collected in Cuatro Ciénegas, Mexico. Samples: water lilies (open circle), substrate (filled square), and snails (open square). Ratios are shown with ± one standard deviation. N = 9 sites for water lilies, N = 10 sites for substrate and snails.

Isotopic signatures varied substantially among sites (Table S5). Average δ^13^C values were lower in the sediment compared to the water lily across populations (paired t = 6.5, df = 8, p>0.001; average difference −5.5‰, standard error 0.8‰). Average δ^15^N values were higher in the sediment compared to the water lily tissue (paired t = −3.3145, df = 8, p-value = 0.01; average difference 3.9±1.2‰). Average δ^13^C values were not significantly different between sediment and snails (paired t = −0.25, df = 9, p-value = 0.81; average difference 0.1±0.5‰). Average δ^15^N values were higher in the snails compared to the sediment (paired t = −5.9262, df = 9, p<0.001; average difference 2.2±0.4‰) (Figure 1). The local isotopic signature of water lily tissue correlated strongly with the signature in the sediment in the case of δ^15^N (r = 0.69, p<0.0001) but not in the case of δ^13^C (r = −0.27, p = 0.74).

### Relationship between Crushing Resistance, Water Lily Abundance, and Other Variables

All factors potentially related to crushing resistance varied substantially among sites (Table S6). Regresssion models that included both pH and snail densities showed VIFs ≥10 because these two variables were highly correlated with each other (r = −0.82, p = 0.01). The partial correlation analysis revealed that snail density contributes more than pH to explain variation in crushing resistance (r = 0.68 vs r = 0.57). In addition, the effect of pH on crushing resistance is negligible when Ca is included (−0.04) despite the correlation between pH and Ca is weak (r = 0.16, p = 0.30). For these reasons, pH was dropped from all regression models to eliminate multicollinearity. The remaining models did not show any evidence of multicollinearity (VIFs <2 in all cases).

The comparison of the remaining multivariate linear regression models showed that crushing resistance is better explained by the combined effects of water lily abundance, the concentration of Ca and P, molariform density and snail density. Models that included several combinations of these terms had AIC scores within less than 4 units from each other, while most models that lacked two or more of those terms showed AIC scores that were more than 9 units higher (Table 1) and therefore have no support [57]. We conducted a similar analysis using the total density of *H. minckleyi* (i.e. papilliforms + molariforms) instead of the density of molariforms and obtained a similar conclusion (Table S7). Partial regression coefficients revealed that the variable that best explained variation in crushing resistance when the other variables were included in the model was water lily abundance (r = 0.84); the contribution of the other variables was substantially lower, (total fish densities: r = 0.58; density of molariform fish: r = 0.56; Ca: r = 0.51; P: r = 0.52; snail density: r = 0.67).

**Table 1:** Competing linear regression models ranked by AIC scores.

Model	AIC	_Δ_AIC
**crushing = Ca+P+molariform+Nymphaea+density**	**52.76**	**0.00**
**crushing = Ca+molariform+Nymphaea**	**55.15**	**2.39**
**crushing = P+Nymphaea**	**55.23**	**2.47**
**crushing = Ca+P+molariform+Nymphaea**	**55.45**	**2.68**
**crushing = Ca+P+Nymphaea**	**55.59**	**2.83**
**crushing = Ca+Nymphaea**	**55.98**	**3.22**
**crushing = P+molariform+Nymphaea**	**56.01**	**3.25**
crushing = Ca+P+molariform	62.21	9.44
crushing = molariform+Nymphaea	62.63	9.87
crushing = molariform	63.19	10.43
crushing = P	65.42	12.66
crushing = Nymphaea	66.01	13.25
crushing = Ca+P+density	67.45	14.68
crushing = Ca	68.31	15.55
crushing = density	73.01	20.25

Variables included in the models: calcium (Ca) and phosphorus (P) concentration, molariform fish density, water lily abundance and snail density. _Δ_AIC is the difference between the AIC values of a given model and the model with the lowest AIC value. Models with statistical support (i.e. _Δ_AIC ≤6) are shown in bold.

**Figure 2:**
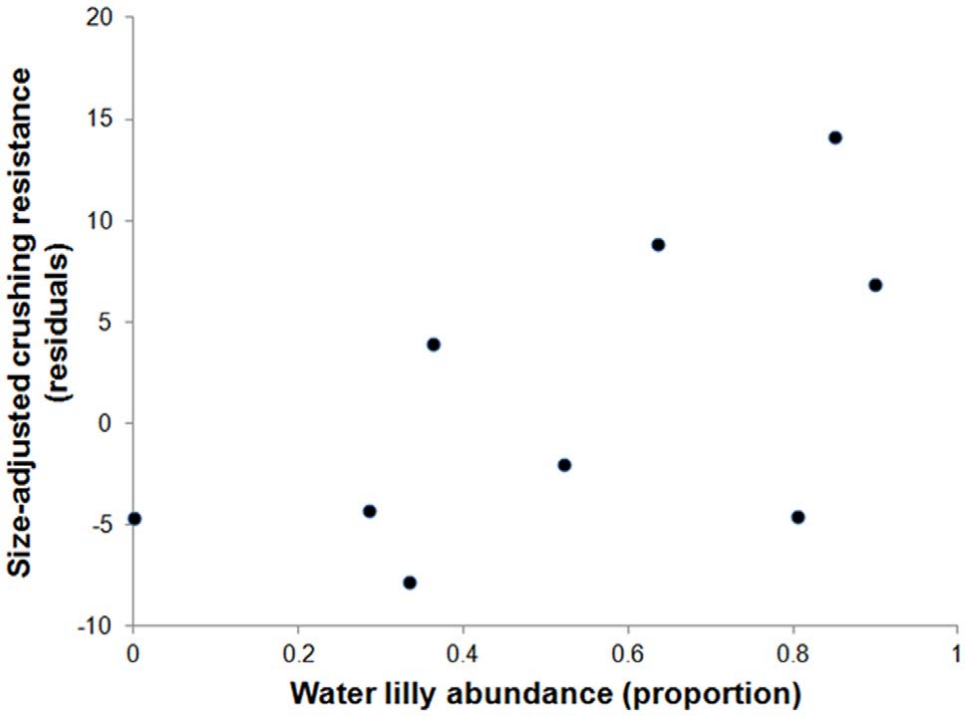
Relationship between water lily abundance and size-adjusted crushing resistance in Cuatro Ciénegas, Mexico. Each point represents a different population (N = 9).

The pairwise correlation analyses (randomization tests) also showed that water lily abundance is better correlated with crushing resistance than any other variable by itself. Average size-adjusted crushing resistance values increased significantly with the local abundance of water lilies (r = 0.58, p = 0.048; Figure 2). Individual correlations between crushing resistance and the other variables were lower and not significant (total fish densities: r = 0.10, p = 0.38; density of molariform fish; r = 0.39, p = 0.16; Ca: r = 0.30, p = 0.22; P: r = 0.54, p = 0.07; snail density: r = 0.46, p = 0.10). In addition, the correlation between P and water lily abundance turned out to be negative (r = −0.20) and not statistically significant (p = 0.30). The correlation between water lily abundance and Ca concentration was nearly zero and not significant (r = −0.04, p = 0.45). Finally, the correlation between snail density and water lily abundance was low and not significant (r = 0.23, p = 0.26).

## Discussion

### Trophic Analysis

The results of the trophic analysis are consistent with the assumption that snails feed on water lily organic matter metabolized by sediment bacteria. The isotopic signature found in the sediment is within the range of what is predicted if sediment bacteria that discriminate against ^13^C were consuming water lily tissue. We found a 5.5‰ decrease in δ^13^C in the sediment in comparison with the water lily, consistent with previous studies on bacteria feeding on detritus [39]. We also found an increase of about 4‰ in δ^15^N between water lily and the sediments, which is a value expected if sediment bacteria were consuming water lily tissue, i.e. one trophic step in this direction [38]. If water lilies were consuming N from the sediment, the ^15^N values for the water lily should have been higher than the values found in the sediment, but this was not the case. Altogether, these values suggest that sediment bacteria are incorporating C and N from water lilies; a flow of C and N in the opposite direction would be relatively small, if existent. Likewise, the isotopic signature of the snail tissue is also in the range of what is predicted if snails were eating sediment bacteria: an average difference in δ^13^C of about 0.5‰ between sediment samples and snail tissue, and an increase of 2.2‰ in δ^15^N between sediment and snail tissue, i.e. one trophic step [38].

These results should be considered with caution, because we cannot discard the possibility that other potential sources of key organic compounds not included in this study, such as side vegetation or algae, have isotopic signatures similar to that of water lilies and are contributing to our results. It is also possible that the isotopic signature found in the sediment is produced *in situ* by chemoautotrophic bacteria that decompose sulphur in the sediment [44] instead of heterotrophic bacteria that feed on water lily organic matter. However, the strong correlation between the local δ^15^N signatures of water lily tissue and local δ^15^N signatures in the sediment suggests that a large proportion of the δ^15^N isotopic signature found in the sediment is the product of metabolization of water lily tissue by heterotrophic bacteria, and that the proportion is similar across sites (one trophic step). Alternatively, the correlation may suggest an uptake of N from the sediment by the lily plant, but the isotopic analysis suggests that most of the N flows from the water lily to the sediment but not in the opposite direction. The lack of correlation in the case of δ^13^C may have been caused by among-site variation in the proportion of bacteria that discriminate against ^13^C versus the proportion of bacteria that does not.

### Correlation between Crushing Resistance and Water Lily Abundance

We found extensive population variation in the abundance of the water lily and shell crushing resistance, and we found that these two variables were positively correlated with each other. Moreover, the analyses suggest that the variation in the abundance of water lily is the best predictor of the variation in crushing resistance. Although the multivariate analyses suggest the concentration of Ca helps explain some of the variation in crushing resistance, the correlation between Ca concentration and crushing resistance turned to be much lower in relation to the correlation between water lily abundance and crushing resistance. The concentration of Ca was very high (around 300 ppm), matching previous reports [42], [43]. This is at least 3 times higher than other freshwater environments, which are usually well below 100 ppm [61], [62]. Hence, Ca does not seem to be a limiting factor in Cuatro Ciénegas, but nonetheless seems to have some effect on crushing resistance. The strong negative correlation found between snail density and pH in conjunction with the positive correlation found between snail density and crushing resistance could help explain the effect of Ca. Altogether, those correlations imply potential higher shell erosion rates in populations with low pH [48], which also tend to have higher densities of snails and stronger shells. This is consistent with a compensatory effect of Ca on shell erosion, which is consistent with recent findings that low pH does not affect calcification rates [47], [48].

In the case of P, the concentration across sites was very low (<0.008 ppm) in agreement with previous reports [42]. These concentrations are considered the lowest reported in continental waters [44], and are expected to affect growth rates in local snails [45] and therefore local crushing resistance [9], [17]. The correlation analysis suggests a potential effect of P on growth rates that may interact with the abundance of local organic compounds to determine the strength of shell material. Given how limited Cuatro Ciénegas is for P [44], the abundance of the water lily may also be affected by the local abundance of P, which in turn may be affected by the abundance of Ca [46]. The correlation between water lily abundance and P concentration was actually negative and clearly not significantly different from zero, as it was in the case of Ca. Our interpretation of these results is that although the abundance of P and Ca in the environment might influence crushing resistance, they are not the cause behind the correlation between water lily abundance and crushing resistance. In addition, the lack of correlation between snail density and water lily abundance, and the fact that the correlation between snail density and crushing resistance was lower than the correlation between the latter and water lily abundance indicates that intraspecific competition is not causing the observed correlation between water lily abundance and crushing resistance. Nevertheless the effect of snail density on growth rates may contribute to some of the variation observed in crushing resistance through intraspecific competition for organic nutrients, and deserves further study.

### Hypothetical Mechanism

Our results are consistent with the hypothesis that the availability of organic matter contributes to shell crushing resistance. Water lily organic matter is assumed to contain organic compounds not synthesized by the snails that are important to produce strong shells. Recent studies on the biomineralization of snail shells show that the small organic fraction found in shells determines the properties of the calcium carbonate crystals in the shell [20] [21] [22] [23], suggesting that the ingestion of at least some organic compounds affects the deposition of calcium carbonate [24]. Hydrobiid snails feed on organic matter and bacteria only, and our finding that shells are relatively weak in areas with few water lilies and relatively hard in areas with many water lilies suggests that the local amount of organic compounds that come from water lilies and are key to the production of shell material may determine, at least partially, shell hardness in a population.

To our knowledge, this is the first evidence that snail shell hardness, and therefore resistance to predation, can be influenced by the abundance of organic compounds in the environment. Numerous studies have demonstrated that shell defenses can be both fixed/constitutive (i.e. do not require environmental stimuli for activation) and/or plastic/induced (i.e. require environmental stimuli) in snails [17], [63], [64]. It is unknown whether shell crushing resistance in *M. churinceanus* is constitutive or induced, but the hypothesis that snails develop stronger shells in environments that are richer in organic matter is potentially compatible with both mechanisms. If shell strength is a constitutive defense, then snails should be selected to grow shells that are as hard as necessary in response to local intensity in predation pressure [65]. The presence of the fish predator (both papilliform and molariforms) has been previously confirmed in all the sites where the snails were collected for at least several decades [29], [30], [66]. The local density of molariforms is not as strongly correlated to local levels of crushing resistance as it is to the density of water lilies. This suggests that high levels of crushing resistance in a population correspond to high level of predation pressure to a lesser extent compared to high abundances of water lilies. Hence, shell thickness should be the product of active calcification more than a byproduct of reduced feeding and somatic growth [9], [16]. Hence, the local amount of key organic compounds, important for shell biomineralization, can determine the content of organic material in shells in a population, determining the mechanical properties of shells, including their hardness [9], [16].

In the case of plastic defenses, the presence of water-born chemicals from predators, and especially from both predators and consumed prey, induce snails to develop thicker shells through reductions in feeding and somatic growth [8], [9], [12], [67], [68]. The process is reversible if the predators disappear [69]. As stated above, the presence of molariform fish has been confirmed in all sites included in this study for several decades. Therefore the snails have been continuously exposed to predation by molariforms and should be permanently induced to produce thicker shells. If the production of thicker shells is only induced by the presence of the predator [67], snails in all populations would be equally induced to produce thicker shells. Under this scenario, the local amount of organic compounds key to shell biomineralization, would determine the organic content in the shells within a population, and therefore their hardness, under either slow or fast somatic growth rates. It is also possible that the snails respond differently to the local amount of chemical cues in the water, i.e. respond accordingly to the magnitude of predation. The density of molariform fish, or of *H. minckleyi* in general, were not the main factor explaining variation in crushing resistance, indicating that differences in crushing resistance found across sites cannot be only attributed to the local concentration of chemical cues, even if each fish morph produced different cues. If the snails respond to the combination of cues from fish and damaged snails [68], then the presence and amount of cues from damaged conspecifics should be correlated with the density of molariforms, which again, is not as strongly correlated with crushing resistance as with the abundance of the water lily. Hence, as in the case of constitutive defenses, shell thickness should be produced by active calcification more than by reduced feeding and somatic growth.

### Future Work

Here we have provided empirical support, although correlational, to a novel hypothesis in the context of resistance to predation in a natural population. We propose for the first time that a third trophic level could be important in the coevolution of prey defensive traits and predatory structures. The next step is to evaluate our hypothesis experimentally, by raising snails in captivity under different levels of organic matter and measuring crushing resistance and organic content in the shell. It should be noted that it is possible that the overall availability of food is shaping shell strength through energetic constraints, rather than the availability of specific organic compounds important for shell biomineralization. This can be tested in the laboratory by feeding snails with diets similar in energetic content but different in organic composition. Unfortunately, we have failed at mimicking the soft sediments in hot and saline spring-fed environments where *M. churinceanus* lives [28], which has excluded the possibility of conducting these experiments ourselves. We encourage other researchers to conduct the necessary experiments with other snail species.

## Supporting Information

Figure S1 **The Cuatro Ciénegas valley in northeastern Mexico showing the ten spring-fed habitats where the samples were collected.** These pools and streams are arrayed around a mountain (Sierra de San Marcos; gray area in the map) that juts into the center of the valley. Three geographic drainages are presented and color coded: western, Río Mesquites, and southeastern. Small black circles represent unsampled pools in the area. The valley is depicted as inset to the image on the right of the map that shows the boundary between Mexico and the United States.
(TIF)

Figure S2 **Relationship between shell length and crushing resistance in** ***Mexipyrgus churinceanus*** **snails.** The best linear regression line, with corresponding equation, is shown. N = 290.
(TIF)

Table S1 **Collection sites in Cuatro Ciénegas, Mexico.**
(DOC)

Table S2 **Comparison of regression models (crushing resistance by site using shell length as covariate) with and without the interaction term (to test for multiple slopes), and with one or multiple intercepts (to test for differences among populations).** The effect of removing the interaction term is negligible in terms of significance (Analysis of Variance) and change in AIC value (_Δ_AIC), whereas the effect of removing the “site” term is large. Hence, crushing resistance scales with shell size in the same way among populations (common slope) but populations vary in crushing resistance for the same shell size (different intercepts).
(DOC)

Table S3 **Average water lily abundance with standard deviation from populations in Cuatro Ciénegas, Mexico.** Abundance is expressed as the proportion of 1×1 m sampled quadrats with water lilies (see text). N = number of transects sampled.
(DOC)

Table S4 **Population size-adjusted crushing resistance with 95% confidence intervals from populations in Cuatro Ciénegas, Mexico.** Average crushing resistance values were calculated for the mid value of the full range of shell length using the R package “effects” (Fox 2003). Abbreviations as in Table S3. N = 30 in all cases except for TC (N = 24), ESC (N = 25), and RM (N = 31). Fox J (2003) Effect displays in R for generalized linear models. Journal of Statistical Software 8: 1–27.
(DOC)

Table S5 **Average stable isotope ratios for C (δ^13^C) and N (δ^15^N) (with standard deviation) measured in water lily tissue, substrate and snail tissue from ten sites in Cuatro Ciénegas, Mexico.** Abbreviations as in Table S3. N = 3 in all cases.
(DOC)

Table S6 **Mean and standard deviation for Calcium (Ca) and phosphorus (P) concentration (ppm), and population fish density (individuals/m^2^) in sampled rivers and pools in Cuatro Ciénegas, Mexico.** Abbreviations as in Table S3 in addition to Pozas Azules (PA). N = 3 in all cases for Ca and P. N = 4 in all cases for fish densities except RM, TB, JS (N = 8) and LR (N = 6).
(DOC)

Table S7 **Competing linear regression models ranked by AIC scores.** Variables included in the models: calcium (Ca) and phosphorus (P) concentration, total fish density (papilliforms + molariforms), water lily abundance, and snail density. _Δ_AIC is the difference between the AIC values of a given model and the model with the lowest AIC value. Models with statistical support (i.e. _Δ_AIC ≤6) are shown in bold.
(DOC)
