# Supplementary figures and images for: The Effect of Aquatic Plant Abundance on Shell Crushing Resistance in a Freshwater Snail
Source: PLoS One. 2012 Sep 6;7(9):e44374. doi: 10.1371/journal.pone.0044374 (PMC3435308; doi:10.1371/journal.pone.0044374)

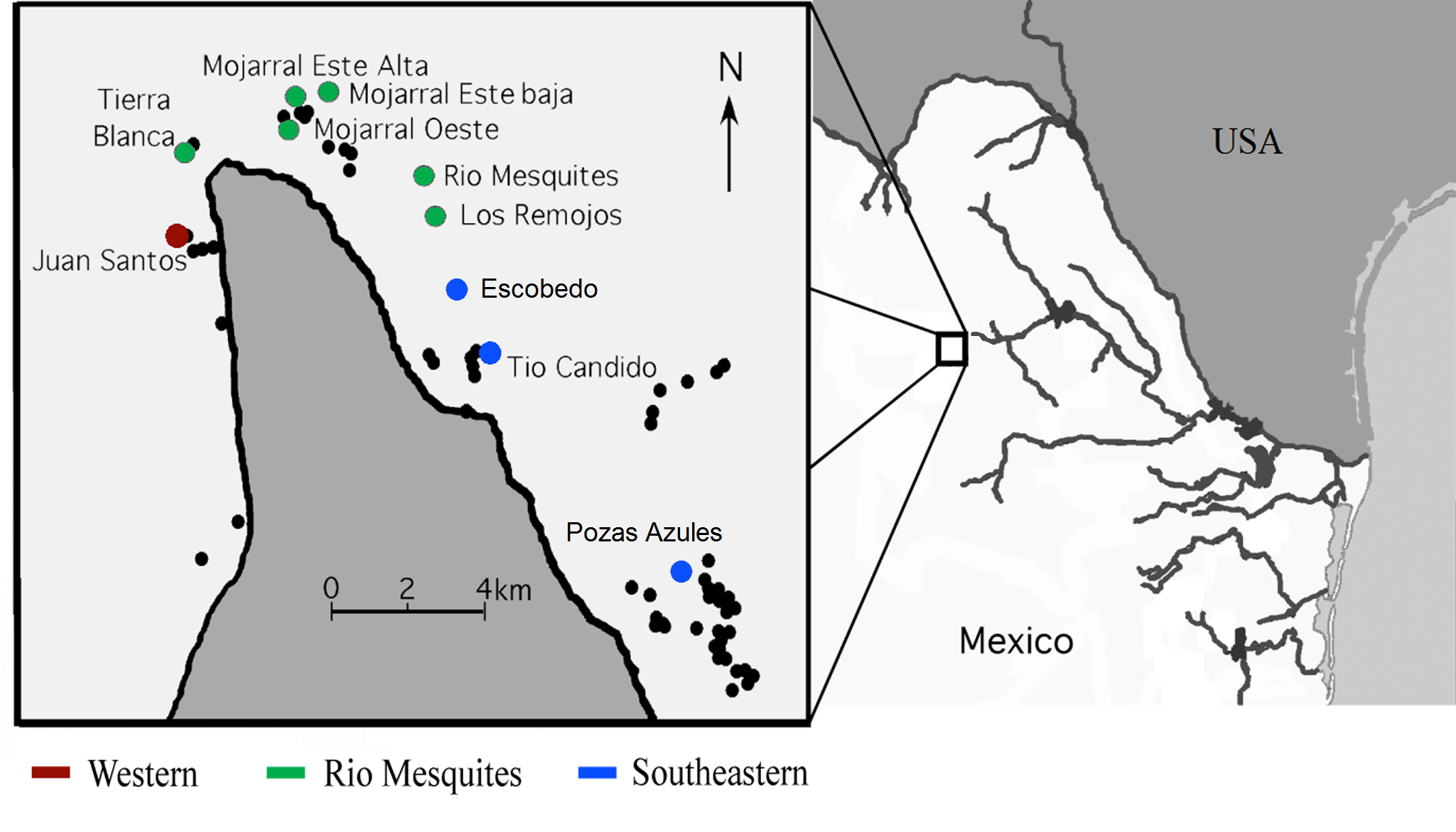

Supplement: Figure S1 — The Cuatro Ciénegas valley in northeastern Mexico showing the ten spring-fed habitats where the samples were collected. These pools and streams are arrayed around a mountain (Sierra de San Marcos; gray area in the map) that juts into the center of the valley. Three geographic drainages are presented and color coded: western, Río Mesquites, and southeastern. Small black circles represent unsampled pools in the area. The valley is depicted as inset to the image on the right of the map that shows the boundary between Mexico and the United States. (TIF) [file pone.0044374.s001.tif]

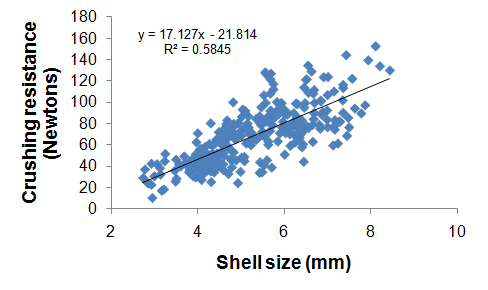

Supplement: Figure S2 — Relationship between shell length and crushing resistance in Mexipyrgus churinceanus snails. The best linear regression line, with corresponding equation, is shown. N = 290. (TIF) [file pone.0044374.s002.tif]
