# Supplementary material for: The Effect of Aquatic Plant Abundance on Shell Crushing Resistance in a Freshwater Snail
Source: PLoS One. 2012 Sep 6;7(9):e44374. doi: 10.1371/journal.pone.0044374 (PMC3435308; doi:10.1371/journal.pone.0044374)
Supplement: Table S1 — Collection sites in Cuatro Ciénegas, Mexico. (DOC) [file pone.0044374.s003.doc]

| **Site** | **GPS coordinates** |
| --- | --- |
| Escobedo | 26°53.59N, 102°05.34W |
| Juan Santos | 26°53.97N, 102°08.96W |
| Mojarral Este Baja (Mojarral Este - East) | 26°55.36N, 102°07.21W |
| Mojarral Este Alta (Mojarral Este - West) | 26 °55.35N, 102 07.06W |
| Mojarral Oeste | 26°55.47N, 102°07.50W |
| Pozas Azules | 26º49.83N, 102º01.76W |
| Río Mesquites | 26°55.47N, 102°06.67W |
| Los Remojos | 26°55.01N, 102°06.67W |
| Tierra Blanca | 26°55.65N, 102°08.31W |
| Tío Cándido | 26°52.33N, 102°04.85W |
