# Supplementary material for: The Effect of Aquatic Plant Abundance on Shell Crushing Resistance in a Freshwater Snail
Source: PLoS One. 2012 Sep 6;7(9):e44374. doi: 10.1371/journal.pone.0044374 (PMC3435308; doi:10.1371/journal.pone.0044374)
Supplement: Table S2 — Comparison of regression models (crushing resistance by site using shell length as covariate) with and without the interaction term (to test for multiple slopes), and with one or multiple intercepts (to test for differences among populations). The effect of removing the interaction term is negligible in terms of significance (Analysis of Variance) and change in AIC value (ΔAIC), whereas the effect of removing the “site” term is large. Hence, crushing resistance scales with shell size in the same way among populations (common slope) but populations vary in crushing resistance for the same shell size (different intercepts). (DOC) [file pone.0044374.s004.doc]

| Model | Factor | df | F | P | AIC | _Δ_AIC |
| --- | --- | --- | --- | --- | --- | --- |
| 1: Different slopes, different intercepts | Size | 1 | 536.9 | <0.001 | 1595.3 |  |
|  | Site | 9 | 10.7 | <0.001 |  |  |
|  | Size x Site | 9 | 1.9 | >0.05 |  |  |
|  | Residual | 269 |  |  |  |  |
|  |  |  |  |  |  |  |
| 2: One slope, different intercepts | Size | 1 | 522.4 | <0.001 | 1594.7 |  |
|  | Site | 9 | 10.4 | <0.001 |  |  |
|  | Residual | 240 |  |  |  |  |
|  |  |  |  |  |  |  |
|  |  |  |  |  |  |  |
| 3: One slope, one intercept | Size | 1 | 403.7 | <0.001 | 1660.4 |  |
|  | Residual | 287 |  |  |  |  |
|  |  |  |  |  |  |  |
| Model 1 vs Model 2 |  | 9 | 1.9 | >0.05 |  | 0.6 |
| Model 2 vs Model 3 |  | 9 | 10.4 | <0.001 |  | 65.7 |
