# Supplementary material for: The Effect of Aquatic Plant Abundance on Shell Crushing Resistance in a Freshwater Snail
Source: PLoS One. 2012 Sep 6;7(9):e44374. doi: 10.1371/journal.pone.0044374 (PMC3435308; doi:10.1371/journal.pone.0044374)
Supplement: Table S3 — Average water lily abundance with standard deviation from populations in Cuatro Ciénegas, Mexico. Abundance is expressed as the proportion of 1×1 m sampled quadrats with water lilies (see text). N = number of transects sampled. (DOC) [file pone.0044374.s005.doc]

| Site | Mean | SD | N |
| --- | --- | --- | --- |
| Escobedo (ESC) | 0.00 | 0.00 | 3 |
| Juan Santos (JS) | 0.64 | 0.15 | 5 |
| Los Remojos (LR) | 0.36 | 0.29 | 3 |
| Mojarral Este-East Side (MEE) | 0.90 | 0.12 | 5 |
| Mojarral Este-West Side (MEW) | 0.66 | 0.16 | 5 |
| Mojarral Oeste (MO) | 0.29 | 0.11 | 3 |
| Río Mesquites (RM) | 0.16 | 0.15 | 5 |
| Tierra Blanca (TB) | 0.84 | 0.12 | 5 |
| Tio Cándido (TC) | 0.92 | 0.14 | 3 |
