# Supplementary material for: The Effect of Aquatic Plant Abundance on Shell Crushing Resistance in a Freshwater Snail
Source: PLoS One. 2012 Sep 6;7(9):e44374. doi: 10.1371/journal.pone.0044374 (PMC3435308; doi:10.1371/journal.pone.0044374)
Supplement: Table S4 — Population size-adjusted crushing resistance with 95% confidence intervals from populations in Cuatro Ciénegas, Mexico. Average crushing resistance values were calculated for the mid value of the full range of shell length using the R package “effects” (Fox 2003). Abbreviations as in Table S3. N = 30 in all cases except for TC (N = 24), ESC (N = 25), and RM (N = 31). Fox J (2003) Effect displays in R for generalized linear models. Journal of Statistical Software 8: 1–27. (DOC) [file pone.0044374.s006.doc]

| Site | Mean | 95% CI |
| --- | --- | --- |
| ESC | 58.52 | 52.0-65.0 |
| JS | 67.78 | 62.2-73.4 |
| LR | 75.15 | 69.4-80.9 |
| MEE | 79.53 | 73.9-85.2 |
| MEW | 71.93 | 66.1-77.8 |
| MO | 58.74 | 52.7-64.8 |
| RM | 61.49 | 56.0-67.0 |
| TB | 87.52 | 81.5-93.6 |
| TC | 69.81 | 63.0-76.6 |
