# Supplementary material for: The Effect of Aquatic Plant Abundance on Shell Crushing Resistance in a Freshwater Snail
Source: PLoS One. 2012 Sep 6;7(9):e44374. doi: 10.1371/journal.pone.0044374 (PMC3435308; doi:10.1371/journal.pone.0044374)
Supplement: Table S5 — Average stable isotope ratios for C (δ13C) and N (δ15N) (with standard deviation) measured in water lily tissue, substrate and snail tissue from ten sites in Cuatro Ciénegas, Mexico. Abbreviations as in Table S3. N = 3 in all cases. (DOC) [file pone.0044374.s007.doc]

| Site | δ^13^C snails | | δ^15^N snails | | δ^13^C water lily | | δ^15^N water lily | | δ^13^C substrate | | δ^15^N substrate | |
| --- | --- | --- | --- | --- | --- | --- | --- | --- | --- | --- | --- | --- |
|  | mean | stdev | mean | stdev | mean | stdev | mean | stdev | mean | stdev | mean | stdev |
| ESC | -27.18 | 0.69 | 11.87 | 0.68 | – | – | – | – | -28.70 | 0.71 | 10.30 | 0.35 |
| JS | -28.05 | 0.03 | 18.62 | 0.91 | -19.71 | 0.13 | 14.97 | 0.14 | -28.64 | 1.15 | 16.10 | 0.45 |
| LR | -27.53 | 0.23 | 16.95 | 0.40 | -21.29 | 0.10 | 14.06 | 0.03 | -28.55 | 0.98 | 15.79 | 0.77 |
| MEE | -26.57 | 0.91 | 17.35 | 1.73 | -22.02 | 0.07 | 12.36 | 0.12 | -25.80 | 0.48 | 14.89 | 0.66 |
| MEW | -27.17 | 0.46 | 13.74 | 0.36 | -20.50 | 0.26 | 13.29 | 0.14 | -27.74 | 1.17 | 12.26 | 0.64 |
| MO | -26.77 | 0.17 | 12.51 | 0.29 | -19.76 | 0.40 | 6.93 | 0.07 | -27.96 | 1.40 | 11.19 | 1.16 |
| PA | -26.75 | 0.29 | 17.26 | 0.34 | -20.57 | 0.05 | 1.57 | 0.02 | -26.28 | 0.86 | 12.49 | 0.34 |
| RM | -25.61 | 0.57 | 17.45 | 0.07 | -21.46 | 0.40 | 6.91 | 0.09 | -23.88 | 0.15 | 13.94 | 0.15 |
| TB | -26.21 | 0.80 | 13.84 | 0.92 | -23.93 | 0.01 | 8.20 | 0.04 | -28.21 | 1.41 | 11.99 | 0.22 |
| TC | -28.96 | 0.28 | 14.48 | 1.48 | -24.30 | 0.08 | 8.74 | 0.06 | -26.21 | 0.85 | 13.23 | 0.63 |
