# Supplementary material for: The Effect of Aquatic Plant Abundance on Shell Crushing Resistance in a Freshwater Snail
Source: PLoS One. 2012 Sep 6;7(9):e44374. doi: 10.1371/journal.pone.0044374 (PMC3435308; doi:10.1371/journal.pone.0044374)
Supplement: Table S6 — Mean and standard deviation for Calcium (Ca) and phosphorus (P) concentration (ppm), and population fish density (individuals/m2) in sampled rivers and pools in Cuatro Ciénegas, Mexico. Abbreviations as in Table S3 in addition to Pozas Azules (PA). N = 3 in all cases for Ca and P. N = 4 in all cases for fish densities except RM, TB, JS (N = 8) and LR (N = 6). (DOC) [file pone.0044374.s008.doc]

|  | Ca | | P | | Fish | |
| --- | --- | --- | --- | --- | --- | --- |
| Site | mean | sd | mean | sd | mean | sd |
| ESC | 339.0 | 59.2 | 0.08 | 0.02 | 3.94 | 0.99 |
| JS | 314.0 | 10.3 | 0.07 | 0.05 | 0.45 | 0.76 |
| MEE | 351.4 | 16.5 | 0.06 | 0.04 | 0.97 | 0.56 |
| MEW | 333.9 | 24.0 | 0.06 | 0.02 | 1.90 | 0.69 |
| MO | 305.3 | 3.2 | 0.02 | 0.01 | 1.08 | 0.34 |
| PA | 328.6 | 41.6 | 0.03 | 0.01 | – | – |
| RM | 389.3 | 24.3 | 0.04 | 0.02 | 0.66 | 0.43 |
| TB | 371.7 | 14.3 | 0.07 | 0.05 | 1.90 | 1.11 |
| TC | 287.3 | 28.4 | 0.02 | 0.00 | 1.18 | 0.86 |
