# Supplementary material for: The Effect of Aquatic Plant Abundance on Shell Crushing Resistance in a Freshwater Snail
Source: PLoS One. 2012 Sep 6;7(9):e44374. doi: 10.1371/journal.pone.0044374 (PMC3435308; doi:10.1371/journal.pone.0044374)
Supplement: Table S7 — Competing linear regression models ranked by AIC scores. Variables included in the models: calcium (Ca) and phosphorus (P) concentration, total fish density (papilliforms + molariforms), water lily abundance, and snail density. ΔAIC is the difference between the AIC values of a given model and the model with the lowest AIC value. Models with statistical support (i.e. ΔAIC ≤6) are shown in bold. (DOC) [file pone.0044374.s009.doc]

| Model | AIC | ΔAIC |
| --- | --- | --- |
| **crushing=Ca+P+fish+Nymphaea+density** | **52.43** | **0.00** |
| **crushing=P+Nymphaea** | **55.23** | **2.80** |
| **crushing=Ca+fish+Nymphaea** | **55.46** | **3.03** |
| **crushing=Ca+P+Nymphaea** | **55.59** | **3.16** |
| **crushing=Ca+Nymphaea** | **55.98** | **3.54** |
| **crushing=Ca+P+fish+Nymphaea** | **56.61** | **4.17** |
| **crushing=P+fish+Nymphaea** | **56.90** | **4.47** |
| crushing=P | 61.00 | 8.56 |
| crushing=Ca+P+fish+density | 61.60 | 9.18 |
| crushing=Ca+P+fish | 62.88 | 10.44 |
| crushing=Nymphaea | 63.19 | 10.76 |
| crushing=fish+Nymphaea | 63.28 | 10.85 |
| crushing=fish | 66.78 | 14.35 |
| crushing=Ca | 68.31 | 15.88 |
| crushing=density | 73.01 | 20.57 |
